# Specific activity of cyclin-dependent kinase I is a new potential predictor of tumour recurrence in stage II colon cancer

**DOI:** 10.1038/bjc.2011.504

**Published:** 2011-11-22

**Authors:** E C M Zeestraten, M Maak, M Shibayama, T Schuster, U Nitsche, T Matsushima, S Nakayama, K Gohda, H Friess, C J H van de Velde, H Ishihara, R Rosenberg, P J K Kuppen, K-P Janssen

**Affiliations:** 1 Department of Surgery, Leiden University Medical Center, 2300 Leiden, The Netherlands; 2 Department of Surgery, Klinikum Rechts der Isar, Technische Universität München, Ismaninger Str. 22, 81675 Munich, Germany; 3 Sysmex Corporation, Central Research Laboratory, Kobe, 651-2271 Hyogo, Japan; 4 Institut für Medizinische Statistik und Epidemiologie, Technische Universität München, 81675 Munich, Germany

**Keywords:** colorectal cancer, metastasis, biomarker, cyclin-dependent kinase, prognosis

## Abstract

**Background:**

There are no established biomarkers to identify tumour recurrence in stage II colon cancer. As shown previously, the enzymatic activity of the cyclin-dependent kinases 1 and 2 (CDK1 and CDK2) predicts outcome in breast cancer. Therefore, we investigated whether CDK activity identifies tumour recurrence in colon cancer.

**Methods:**

In all, 254 patients with completely resected (R0) UICC stage II colon cancer were analysed retrospectively from two independent cohorts from Munich (Germany) and Leiden (Netherlands). None of the patients received adjuvant treatment. Development of distant metastasis was observed in 27 patients (median follow-up: 86 months). Protein expression and activity of CDKs were measured on fresh-frozen tumour samples.

**Results:**

Specific activity (SA) of CDK1 (CDK1SA), but not CDK2, significantly predicted distant metastasis (concordance index=0.69, 95% confidence interval (CI): 0.55–0.79, *P*=0.036). Cutoff derivation by maximum log-rank statistics yielded a threshold of CDK1SA at 11 (SA units, *P*=0.029). Accordingly, 59% of patients were classified as high-risk (CDK1SA ⩾11). Cox proportional hazard analysis revealed CDK1SA as independent prognostic variable (hazard ratio=6.2, 95% CI: 1.44–26.9, *P*=0.012). Moreover, CKD1SA was significantly elevated in microsatellite-stable tumours.

**Conclusion:**

Specific activity of CDK1 is a promising biomarker for metastasis risk in stage II colon cancer.

Each year >1 million individuals worldwide develop colon cancer with a disease-specific mortality rate of almost 33% (Parkin *et al*, 2005; Ferlay *et al*, 2010). Approximately 40% of resected colon cancers are from stage II (T3–4N0M0). The 5-year survival rates vary between 88% in T3N0 patients, and 75% in T4N0 patients. Chemotherapy is widely accepted as adjuvant treatment for stage III patients, whose 5-year survival (stage III A and B) is >75% (Gunderson *et al*, 2010). Use of chemotherapy for stage II, T4 patients remains controversial despite their worse survival rates. This indicates that the allocation of treatment based solitary on conventional staging methods is not optimal (Kahlenberg *et al*, 2003; Gunderson *et al*, 2004; Roukos *et al*, 2007; Kozak and Moody, 2008; Poston *et al*, 2008). Over the last decade, there have been important developments towards the discovery of new prognostic and predictive markers that might improve staging methods. The American Society of Clinical Oncology’s Tumor Markers Expert Panel (ASCO TEMP-2006) and its European counterpart, the European Group on Tumor Markers (EGTM-2007) have recently reviewed the literature on these biomarkers. However, all biomarkers reviewed lacked the significant, discriminative value that is required to become implemented into clinical practice (Duffy *et al*, 2003, 2007; Locker *et al*, 2006). There is a stringent need for new assays that are able to identify stage II colon cancer patients who might benefit from adjuvant therapy. Genomic instability and altered cell proliferation are major contributors to tumour growth and aggressiveness. Measuring these hallmarks of colon cancer in a quantitative fashion could be a suitable option for risk stratification. The proliferation rate of tumour cells has so far been studied with methods such as ^3^H-thymidine/BrdU incorporation, mitotic index, or Ki-67/PCNA immunohistochemistry, but none of these tests has reached clinical application (Daidone and Silvestrini, 2001; Michels *et al*, 2004). Therefore, analysis of the highly conserved drivers of the cell cycle, the cyclin-dependent kinases (CDKs) 1 and 2, may be a more promising approach (Malumbres and Barbacid, 2009). Cyclin-dependent kinase expression is constitutive in tumours but their enzymatic activity changes markedly according to the specific cell-cycle phase. On the molecular level, the activity of CDK is regulated by subunits known as cyclins, and by phosphorylation of conserved tyrosine and threonine residues. Over-expression of cyclins, as well as inactivation of CDK inhibitors, are well documented as prognostic markers for oesophageal, gastric, colorectal, breast, and lung cancer (Gansauge *et al*, 1997; Sutter *et al*, 1997; van Diest *et al*, 1997; Murakami *et al*, 1999; Sjostrom *et al*, 2000; Soria *et al*, 2000; Takano *et al*, 2000; Takemasa *et al*, 2000; Korenaga *et al*, 2002; Peters *et al*, 2004; Yoshida *et al*, 2004; Ishihara *et al*, 2005; Boutros *et al*, 2007; Suzuki *et al*, 2007; Begnami *et al*, 2010; Pereg *et al*, 2010; Timofeev *et al*, 2010). However, expression analysis of cyclins and other factors may not necessarily indicate the enzymatic activity of CDKs, which is crucial for the cell-cycle status of the cancer cells. We have recently reported an assay that measures the specific activity (SA) of CDK 1 and CDK2 (Ishihara *et al*, 2005; Kim *et al*, 2008; van Nes *et al*, 2009), based on a well-standardised biochemical assay that requires only small amounts of fresh-frozen tissue and is described in Ishihara *et al* (2005). The hallmark of this approach is the extraction of functional CDK enzyme from tumour tissue, followed by determination of its kinase activity. We hypothesise that intratumoural kinase activity of CDKs predicts the prognosis of tumour patients with great fidelity, because it directly represents a quantifiable readout for two hallmarks of tumours: increased proliferation and genomic instability. Two large, independent cohorts of breast cancer patients demonstrated that this assay had prognostic value (Kim *et al*, 2008; van Nes *et al*, 2009). A CDK-based risk score validated in these studies was a significant and independent prognostic factor, especially for distant recurrence. The aim of this study was to determine the ability of CDK-based analysis to predict recurrence in patients with locally restricted colon cancer. The study was carried out retrospectively on two independent patient cohorts derived from large surgical oncology centres in the Netherlands and Germany. Our results demonstrate that the SA of CDK1 identifies stage II colon cancer patients with a high risk of distant disease recurrence. This patient group may benefit from adjuvant chemotherapy, which would not be recommended according to standard criteria.

## Patients and methods

### Patients

The study was approved by the local ethics committees at LUMC and TUM. Informed, written consent had been obtained before the study. Fresh-frozen samples of 271 of stage II colon carcinomas were analysed, collected at Leiden University Medical Center (LUMC, 1985–2005), and at Klinikum Rechts der Isar (TUM, 1987–2006). All patients had curative (R0) tumour resection, and none of them received adjuvant or neoadjuvant therapy. Tumour tissue was dissected immediately after resection by a pathologist, snap frozen in liquid nitrogen and stored at −80°C. Development of distant metastasis was observed in 27 patients (11%) after a follow-up of 7.2 years (median). Five samples (1.8%) were excluded due to tumour cell content of <10%. All remaining tissue samples underwent C2P analysis, 12 cases were excluded due to assay failure, or CDK expression level below detection threshold (*n*=3). Of note, all 12 excluded cases were free of tumour recurrence. Hence, 254 samples were available for further analysis (*n*=217 from TUM, and *n*=37 from LUMC).

### Determination of CDK-specific activities

In all, 10–20 sections of 100 *μ*m thickness were cut with a cryostat and subjected to CDK analysis. One section of 7 *μ*m thickness was cut from the middle of each block and evaluated by a pathologist after standard H&E staining. Cases with tumour cell content <10% were excluded. The system to measure the CDK-specific activity (CDKSA) is called ‘C2P’ (for ‘cell-cycle profiling’ Sysmex, Kobe, Japan; Ishihara *et al*, 2005; Kim *et al*, 2008). In brief, lysates of frozen material were applied to a well of 96-well PVDF filter plate (Millipore, Billerica, MD, USA). Expression of CDKs was detected quantitatively by sequential reactions with primary anti-CDK antibodies, biotinylated anti-rabbit antibodies, and fluorescein-labelled streptavidin. To measure the kinase activity, CDK molecules were immunoprecipitated from the lysate using protein beads, as reported in detail earlier (Ishihara *et al*, 2005; Kim *et al*, 2008). Cyclin-dependent kinase SA was calculated as CDK kinase activity units (aU *μ*l^–1^ lysate) divided by its corresponding CDK expression units (eU *μ*l^–1^ lysate). Both aU (CDK *activity unit*) and eU (CDK *expression unit*) were defined as the expression and activity equivalent to 1 ng of recombinant CDK1 and CDK2, respectively. The distribution of the CDK1SA and CDK2SA within the LUMC and the TUM cohort can be found in Supplementary Figure 1. Further details regarding the quality controls for this assay can be found in the Supplementary data.

### Immunofluorescence analysis

Tissue specimens (7 *μ*m) from 207 samples were available for evaluation by immunofluorescence microscopy (Axiovert 200, Zeiss, Göttingen, Germany). After fixation with 3% PFA and antigen retrieval (10 min boiling, sodium citrate buffer, pH=6.0), slides were incubated with anti-Ki-67 antibody (clone MIB-1, M 7240; DAKO, Hamburg, Germany) and/or anti-cytokeratin-20 antibody (rabbit monoclonal, 2039-1, Epitomics, Burlingame, CA, USA) diluted 1:200, followed by incubation with secondary antibodies (Molecular Probes, Darmstadt, Germany; Dianova, Hamburg, Germany), and counterstaining with 4′,6′-diamidino-2-phenylindole (DAPI; Invitrogen, Darmstadt, Germany). Ki-67-positive nuclei from CK20-positive cells were regarded as *bona fide* tumour cells and were counted in a semi-automated manner using ImageJ freeware (NIH, Bethesda, MD, USA; http://rsb.info.nih.gov/ij/).

### Microsatellite instability determination

Tissue from 200 patients of the Munich cohort and all 37 patients of the LUMC was available for DNA isolation with the QIAampDNAMini Kit (Qiagen, Hilden, Germany) according to the manufacturer’s protocol. DNA concentration and quality was checked with an ND-1000 NanoDrop Spectrophotometer (Thermo Fisher, Schwerte, Germany). Subsequently, microsatellite instability (MSI) was tested with the MSI Analysis System, Version 1.2 (Promega, Mannheim, Germany). This assay co-amplifies five mononucleotide repeat markers; BAT-25, BAT-26, NR-21, NR-24, and MONO-27 to determine MSI status. It includes two pentanucleotide repeats, Penta C and D, to make sure that normal and tumour samples are derived from the same patient. The results of this assay have been previously compared with the Bethesda panel markers and proven highly sensitive for MSI determination (Murphy *et al*, 2006). The MSI status was determined for 32 of the 37 LUMC cases, and for 191 of 200 TUM patients. In 6% of the cases (14 of 237 available DNA samples), MSI status could not be determined based on evaluation of the PCR array data by an experienced pathologist due to ambiguous results.

### BRAF

The mutational status of the oncogene BRAF (V600E, GTG>GAG substitution in exon 15) was assessed by high-resolution melting analysis of genomic DNA on a Lightcycler 480 II platform (Roche, Mannheim; SYBR Green I/HRM Dye Protocol), in a modification of published protocols (Pichler *et al*, 2009). Briefly, 20 ng of genomic DNA (10 ng *μ*l^–1^) were amplified in total volume of 20 *μ*l with 10 *μ*l High-Resolution Master Mix, 2.4 mM MgCl_2_, and 0.25 mM each of oligonucleotide primers, 2 *μ*l template DNA and 5.2 *μ*l dH_2_O. Primer sequences were BRAF Exon 15 For: 5′-GGT GAT TTT GGT CTA GCT ACA G-3′, BRAF Exon 15 Rev: 5′-AGT AAC TCA GCA GCA TCT CAG G-3′. After pre-incubation (95°C, 10 min), amplification of a 147-bp product was carried out in 42 cycles (95°C, 15 s/61°C, 15 s/72°C, 15 s), followed by melting point analysis with an initial phase: 95°C, 5 s, and 72°C, 90 s, followed by a melting profile ranging from 72°C to 95°C in 19.2 min. As a positive control, genomic DNA from the BRAF-mutated colon cancer cell line HT29 was used.

### Statistical analysis

Statistical analyses were conducted using R Software version 2.11.1 (R Foundation for Statistical Computing, Vienna, Austria). In order to derive optimal cutoff values of quantitative CDK measurements for recurrence risk stratification, maximally selected log-rank statistics have been used. To consider multiple test issue within these analyses, the R-function ‘maxstat.test’ was employed (Hothorn and Zeileis, 2008). To internally validate the derived cutoff, the entire data set was randomly divided in a training and test set (ratio 70 : 30). Furthermore, bootstrap re-sampling analysis was conducted to estimate distribution of derived cutoff values and 95% confidence intervals (CIs), respectively. Multivariable Cox regression was performed to assess recurrence risk differences between derived subgroups in simultaneous consideration of potential confounding factors. Because of the low number of critical events, multivariable regression analyses had to be performed consecutively (one-by-one inclusion of potential confounding factors) to avoid over-adjustment. By the use of survival receiver operating characteristic (ROC) analysis, predictive capability of recurrence risk stratification was assessed cumulatively over the course of the follow-up. In this term, area under the time-dependent ROC curve (concordance index) was reported with 95% bootstrap CI. The Kaplan–Meier methods were used for survival plotting and log-rank test for comparison of survival curves. All statistical tests were conducted two-sided and a *P*-value <0.05 was considered significant.

## Results

We have determined the SA of CDK1 and CDK2 (CDK1SA and CDK2SA) in a study population comprised of samples from two independent cohorts of stage II colon cancer patients originating from the Leiden University Medical Centre (LUMC, The Netherlands) and the Klinikum Rechts der Isar, of the Technical University in Munich (TUM, Germany). Five samples (1.8%) were excluded due to tumour cell content of <10%. Twelve cases were excluded due to assay failure, and in three cases the CDK expression levels were below the detection threshold. Of note, all excluded cases were free of tumour recurrence. Altogether, the expression and kinase assay (‘C2P’, in short for ‘cell-cycle profiling’) yielded results in 96% of patients (254 out of 266; *n*=217 from TUM, and *n*=37 from LUMC). There were no statistically significant differences in clinico-pathological characteristics between both cohorts (Table 1). The SA was calculated and indicated as kinase activity in relation to its corresponding mass concentration. The CDK activity unit and CDK expression unit were defined as the equivalent of 1 ng recombinant CDK protein. The distribution of the CDK1SA did not vary significantly between the two study cohorts (Mann–Whitney *U*-test, *P*=0.35), whereas the average of CDK2SA was higher in samples from the Netherlands (*P*=0.012) (Supplementary Figure 1).

### Predictive performance and cutoff derivation of CDKSA for distant recurrence

**Figure 1:**
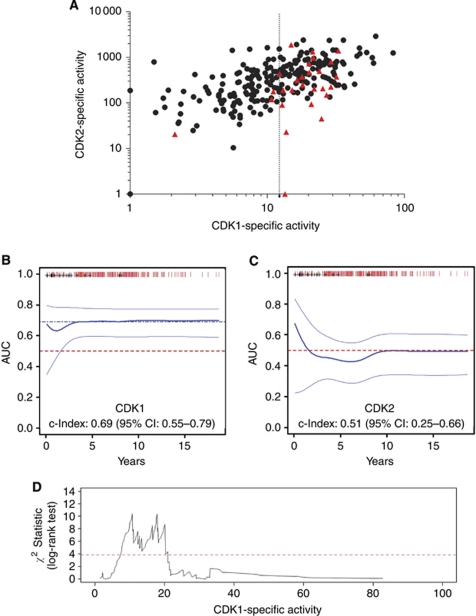
Prognostic performance of the specific activities of CDK1 and CDK2. (**A**) All cases (*n*=254) plotted on a scatter diagram with logarithmic scales according to CDK1SA and CDK2SA, respectively. Red symbols: patient with distant metastasis, black symbol: no metastasis. (**B** and **C**) Time-dependent ROC analysis against CDK1SA (**B**) or CDK2SA (**C**). Thick line: concordance index, thin line: 95% CI. Concordance index was 0.69 for CDK1SA (95% CI: 0.55–0.79, *P*=0.036), and 0.51 for CDK2SA (95% CI: 0.25–0.66, *P*=0.57). (**D**) Derivation of an optimal CDK1SA cutoff value. The maximum log-rank test statistic was obtained when CDK1SA was 11 or 18 (maU eU^–1^).

The distribution of clinical samples was plotted on a scatter diagram according to CDK1SA and CDK2SA (Figure 1A). Cases with distant metastasis clustered in the region with high CDK1 activity, suggesting that mainly CDK1SA could have prognostic power. In order to evaluate the prognostic performance of CDK activity for distant metastasis risk, the true positive rates of distant disease recurrence (sensitivity) and corresponding false positive rates (100-specificity) were summarised in a time-dependent ROC curve. The average area under the ROC curve (concordance index or *AUC*) was 0.69 for CDK1SA (95% CI: 0.55–0.79, *P*=0.024), and 0.51 for CDK2SA, respectively (95% CI: 0.29–0.66, *P*=0.57) (Figures 1B and C). Combined, these results suggested that CDK1SA, but not CDK2SA, is valuable for long-term distant recurrence prediction. Therefore, we focused on CDK1SA and derived the statistically best discriminating cutoff value for CDK1SA, as indicated by maximum log-rank test. For 254 cases, two local maxima of log-rank test statistic were obtained, one for CDK1SA=11 (milli-activity unit per expression unit, maU eU^–1^), and one for CDK1SA=18 (maU eU^–1^) (Figure 1D). In order to test the robustness of the selected cutoff values, a second cutoff derivation was performed using the subset of samples with CDK1SA>11 (maU eU^–1^) (*n*=150). In this analysis, the previously proposed cutoff value of 18 (maU eU^–1^) neither showed a significant maximum peak, nor was considerably elevated compared with the other candidate cutoff values. This result suggested that the optimal cutoff value for CDK1SA was indeed at 11 (maU eU^–1^). The final bootstrap analysis confirmed a cutoff value for CDK1SA of 11 (maU eU^–1^) to be of sufficient discriminant value for further analysis (Supplementary Figure 2). In conclusion, patients with CDK1 activity level >11 (maU eU^–1^) were classified in the high-risk group (*n*=104, 40% of the patients), and the remaining patients as low-risk group (*n*=150, 60%).

### CDK1-based risk prediction for distant metastasis-free survival and cause-specific survival

**Figure 2:**
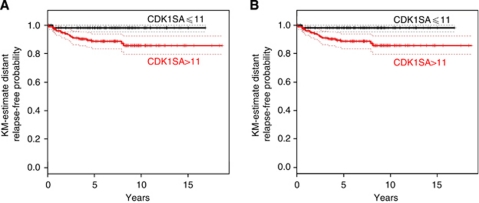
Analysis of distant metastasis-free survival and cause-specific survival. (**A**) Patients classified in the high-risk group (based on CDK1SA >11 maU eU^–1^) had a significantly worse distant metastasis event rate as compared with the low-risk group (HR=6.2, 95% CI: 1.45–26.9, *P*=0.0049; exact conditional Monte-Carlo *P*-value=0.029). (**B**) Patients classified in the CDK1SA-based high-risk group had a significantly lower cause-specific survival (HR=7.62, 95% CI: 1.80–32.2, *P*=0.001).

Univariable ‘time-to-event’ analysis showed that patients from the CDK1SA-based low-risk group had significantly longer distant metastasis-free intervals than patients in the high-risk group (hazard ratio (HR)=6.2, 95% CI: 1.45–26.9, *P*=0.0049) (Figure 2A). Importantly, this finding was retained to be statistically significant after adjusting for the multiple log-rank testing, which had been performed in order to obtain the optimal cutoff value of 11 (maU eU^–1^) (exact conditional Monte-Carlo *P*-value=0.029). The independence of prognostic ability of CDK1SA-based recurrence risk stratification was further evaluated and finally confirmed by multivariable analyses (Table 2). Hazard ratio estimates remained nearly unchanged after consecutive adjustment for the most important clinical-pathological variables, which are currently used for risk evaluation in stage II colon cancer: T4, poor differentiation, presence of obstruction or perforation, lymphatic and vessel invasion, high CEA level, and ⩽12 regional lymph nodes examined (Van Cutsem and Oliveira, 2009) (Table 2). Next, a putative confounding influence of mutations in the BRAF oncogene were analysed. In 217 patients, tissue was available for high-resolution melting analysis of mutations in exon 15 of BRAF. In 32 cases (14.8%), BRAF^V600E^ mutations were detected, 183 patients had BRAF wild-type status, and two cases were not informative. In Kaplan–Meier analysis, the BRAF mutation status was not significantly associated with metastasis-free survival (*P*=0.337), nor with cause-specific survival (*P*=0.253; not shown), and it was excluded as confounding factor for CDK1SA-based risk prediction (Table 2).

However, when considering stroma content as adjustment variable, a lack of statistical significance was apparent for the effect of dichotomised CDK1SA. The apparent absence of significance may be explained by the reduced statistical power for this parameter, since about 30% of the cases lacked available stroma content data. Twenty-five patients (10%) died during the follow-up, among them were all 20 patients with distant metastases, and only five patients with no evidence for distant metastases, but with local tumour recurrence. Because of this strong association of distant relapse and death, CDK1SA categorisation was found to be a significant predictor for cause-specific survival (HR high-risk *vs* low-risk group: 7.62, 95% CI: 1.80–32.2, *P*=0.001) (Figure 2B). This result was thoroughly confirmed in the multivariable analyses. All adjusted estimates of the HR showed values of >7.75, with lower 95% confidence limits >1.80, and *P*-values <0.01. However, a non-significant HR was estimated after adjustment for stroma content (HR high-risk *vs* low-risk group: 5.22, 95% CI: 0.65–41.5, *P*=0.12).

### Correlation between CDKSA, cell proliferation, and microsatellite status

**Figure 3:**
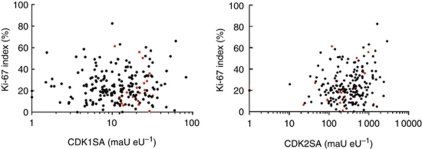
Correlation between CDKSAs and Ki-67 index (percent of Ki-67-positive cells of all CK20-positive tumour cells). Cases were plotted on a scatter diagram according to Ki-67 index against CDK1SA (left), or CDK2SA (right). Red circle: tumour with distant metastasis. Ki-67 showed a weak but significant positive correlation with CDK2SA (Spearman’s *ρ*=0.17, *P*=0.016), but not with CDK1SA (Spearman’s *ρ*=0.04, *P*=0.54).

Based on the knowledge of the process of tumourigenesis, high CDK1SA levels could be a reflection of strongly elevated tumour cell proliferation rates. Therefore, we have analysed tumour cell proliferation with the established proliferation marker Ki-67. The Ki-67 labelling index, defined as the percentage of cytokeratin-20-positive cancer cells with Ki-67-positive nuclei, was determined for *n*=207 cases. The median of the Ki-67 index was 21.4%, but it was not retained by Cox regression analysis as significant prognostic factor for distant metastasis (HR=0.69, 95% CI: 0.02–24.0, *P*=0.84). Next, a putative correlation between CDKSAs and the Ki-67 index was examined. However, no significant correlation was found between CDK1SA and Ki-67 index (Spearman’s *ρ*=0.04, *P*=0.54) (Figure 3).

**Figure 4:**
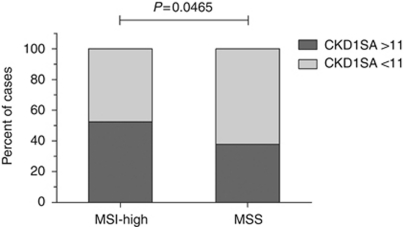
Association of CDK1SA-based risk stratification with microsatellite-stable phenotype. Among the patients with a stable microsatellite phenotype (MSS), 62% (102 out of 164) were classified in the high-risk group based on CDK1SA. On the other hand, 47.5% (28 out of 59) of the patients with high MSI (MSI-H) were classified as high-risk, based on the CDK1SA threshold (*χ*^2^-test, *P*=0.0465).

Lastly, a putative correlation between genomic instability and CDK1 activity was tested, since CDKs have been shown to be implicated in cellular responses to genetic instability. Microsatellite instability, caused by defects in the cellular mismatch repair system, has been suggested for colorectal cancer as a favourable prognostic marker. The MSI status was determined with standard methods for 223 cases, and a high level of instability was detected in 59 tumours (26.5%, MSI-High), whereas 164 samples showed stable microsatellite repeats (73%, MSS). Cox regression analysis indicated an estimated five-fold risk-difference regarding distant metastasis-free survival for microsatellite-stable patients, but the results did not attain significance (HR=5.898, 95% CI: 0.782–44.481, *P*=0.085) (Supplementary Figure 3). A significant association of MSI and CDK1SA-based risk stratification was apparent, based on the cutoff for CDK1SA of 11 (maU eU^–1^). In the patient group with stable microsatellites, significantly more cases with elevated CDK1SA were observed (*χ*^2^-test, *P*=0.0465; Figure 4). However, a direct comparison of CDK1SA between patients with stable or unstable microsatellites did not attain significance (Supplementary Figure 4).

## Discussion

This study is the first report demonstrating the SA of CDK1 (CKD1SA) as prognostic biomarker for stage II colon cancer in a blinded and retrospective manner. Two patient cohorts from Germany and the Netherlands were included in this study. Essentially, no differences were observed between these cohorts regarding clinical parameters or CDK1 activity, indicating that the patients were recruited in an unbiased manner. However, the average of CDK2SA was slightly but significantly higher in the samples from the Netherlands (Supplementary Figure 1). This may be due to differences in sample embedding and preparation between the study centres, and to technical variations between the assay systems for CDK1SA and CDK2SA. Previously, CDK1SA- and CDK2SA-based risk was shown to be a clinically useful prognostic marker of early breast cancer of Caucasian and Asian cohorts (Ishihara *et al*, 2005; Kim *et al*, 2008; van Nes *et al*, 2009).

To identify patients with unfavourable prognosis who might benefit from adjuvant chemotherapy, several types of staging systems have been developed (Astler and Coller, 1954; O’Connell *et al*, 2004; Kozak and Moody, 2008; Gunderson *et al*, 2010). The current staging systems, however, do not provide accurate risk assessment for stage II patients (O’Connell *et al*, 2004; Van Cutsem and Oliveira, 2009). Moreover, a number of molecular markers have been proposed, such as mutations in KRAS and TP53, loss of heterozygosity of chromosome 18, and MSI (Fearon and Vogelstein, 1990; Locker *et al*, 2006; Tejpar *et al*, 2010). However, none of these candidate biomarkers has yet clearly proven to be useful for diagnosis or staging of patients with stage II colorectal cancer, except for mutations in the BRAF oncogene, which were found to be prognostic for overall survival, particularly in patients with microsatellite-stable tumours (French *et al*, 2008; Roth *et al*, 2010). Comprehensive approaches using ‘omics’ technologies have been applied to find biomarkers for colorectal cancer, and we and many others have proposed prognostic transcriptome profile sets so far (Arango *et al*, 2005; Barrier *et al*, 2007; Lin *et al*, 2007; Salazar *et al*, 2010; Webber *et al*, 2010). However, inter-patient and even intratumoural heterogeneity, as well as cost factors have precluded wide-scale clinical application. A promising strategy to circumvent tumour heterogeneity is to focus on the central hallmarks of cancer, which are present in almost all tumours irrespective of the underlying molecular changes. Altered cell proliferation and genomic instability are central hallmarks in the case of colon cancer (Hanahan and Weinberg, 2000; Malumbres and Barbacid, 2009). Therefore, we focused on the enzymatic activities and protein expression of CDKs, the main drivers of cell-cycle progression. Moreover, CDK regulators have been well documented as prognostic indicators in many solid tumours (Gansauge *et al*, 1997; Sutter *et al*, 1997; van Diest *et al*, 1997; Murakami *et al*, 1999; Sjostrom *et al*, 2000; Soria *et al*, 2000; Takano *et al*, 2000; Takemasa *et al*, 2000; Korenaga *et al*, 2002; Peters *et al*, 2004; Yoshida *et al*, 2004; Ishihara *et al*, 2005; Boutros *et al*, 2007; Suzuki *et al*, 2007; Begnami *et al*, 2010; Pereg *et al*, 2010; Timofeev *et al*, 2010)

Indeed, CDK1SA was a substantial and constant marker for long-term event prediction of distant metastasis in the present study. A robust cutoff value for CDK1SA was derived by choosing a threshold with maximum log-rank statistics (Hothorn and Zeileis, 2008). Importantly, the cutoff value of 11 (maU eU^–1^) was verified by the adjusted multiple log-rank test. Multivariate analysis retained CDK1SA as independent predictor of distant recurrence. None of the currently accepted clinical risk factors, for example, T4 stage, poor differentiation, obstruction, or tumour perforation (Van Cutsem and Oliveira, 2009), was identified as confounding factor (Table 2). Moreover, CDK1SA was independent of the mutation status in the BRAF oncogene. Therefore, we conclude that CDK1SA-based risk stratification is a reliable prognostic marker for distant metastasis in stage II colon cancer. Two hypotheses, which are not mutually exclusive, may explain the increased intratumoural CDK1SA level in patients with worse prognosis. First, SA of CDK1 may directly reflect higher cancer cell proliferation. To address this question, we have examined a putative correlation between CDK1SA and proliferation. The index of proliferating cancer cells did not significantly correlate with CDK1SA. Moreover, the Ki-67 proliferation index itself was not significant for prognosis, in accordance with earlier findings (Brown and Gatter, 2002). Second, CDK1 activity may be elevated due to chromosomal instability (CIN), a factor already associated with worse prognosis (Walther *et al*, 2008). Indeed, high CDK1SA levels were significantly correlated with a stable microsatellite phenotype (*χ*^2^-test, *P*=0.0465). To the best of our knowledge, no reports exist that provide a cause-and-effect link between CDK1 activity and MSI. However, colorectal tumours with stable microsatellites are thought to present CIN, associated with worse prognosis. Thus, microsatellite-stable tumours with high CDK1SA levels in our collective are likely to display chromosomal instability. On the molecular level, regulation of CDK1 activity is orchestrated by cellular checkpoints. Altered expression and activity of the DNA damage and spindle-checkpoint proteins are frequently observed in cancer cells, and contribute to chromosomal instability (Malumbres and Barbacid, 2009). Thus, deregulated checkpoint pathways could cause an aberrant activation of CDK1. Indeed, over-expression of both cyclinB1 and CDC25, important regulators of CDK1 activity, are prognostic markers in colorectal and other cancers (Takemasa *et al*, 2000; Korenaga *et al*, 2002; Suzuki *et al*, 2007). In conclusion, CDK1SA-based analysis is a robust and useful assay to identify patients with a high risk of distant recurrence, who could benefit from adjuvant chemotherapy.

## Figures and Tables

**Table 1:** Patient characteristics

**Category**	**Subcategory**	**Total collective (%)**	**Patients from TUM (%)**	**Patients from LUMC** **(%)**
Total		254 (100%)	217 (100%)	37 (100%)
Sex	Male	141 (56)	124 (57)	17 (46)
	Female	113 (44)	93 (43)	20 (54)
Age		65 (Median) 15–91 (range)	65 (Median) 15–91 (range)	69 (Median) 26–82 (range)
Open surgery		254 (100)	217 (100)	37 (100)
Location	Caecum	40 (16)	31 (14)	9 (24)
	Ascending colon	66 (26)	59 (27)	7 (19)
	Transverse colon	26 (10)	23 (11)	3 (8)
	Descending colon	30 (12)	28 (13)	2 (5)
	Sigmoid	92 (36)	76 (35)	16 (43)
Tumour size		6 (Median) 2–15 (range)	6 (Median) 2–15 (range)	5 (Median) 3–14 (range)
pT	T3	221 (87)	188 (87)	33 (89)
	T4	33 (13)	29 (13)	4 (11)
Lymph nodes total		19 (Median) 1–72 (range)	20 (Median) 7–72 (range)	10 (Median) 1–26 (range)
Grading	G1, G2	170 (67)	149 (69)	21 (57)
	G3, G4	77 (30)	65 (30)	12 (32)
	Missing	7 (3)	3 (1)	4 (11)
Recurrence	None	220 (87)	191 (88)	29 (78)
	Distant	27 (11)	22 (10)	5 (14)
	Local	7 (3)	4 (2)	3 (8)
Survival information	Alive	172 (68)	155 (71)	17 (46)
	Tumour-related death	25 (10)	19 (9)	6 (16)
	Non-tumour-related death	58 (23)	44 (20)	14 (38)

Abbreviation: pT=tumour stage.

**Table 2:** Consecutive (one-by-one) adjustment for confounding factors

**Pairwise comparison**	**Category**	**Subcategory**	**HR**	**95% CI for HR lower/upper**		***P*-value**
1	CDK1SA	>11 *vs* ⩽11	4.23	0.52	34.11	**0.180** a
	Stroma content		1.02	0.99	1.04	0.230
2	CDK1SA	>11 *vs* ⩽11	6.24	1.44	26.93	**0.014**
	Histol. grade	>2 *vs* ⩽2	0.99	0.13	7.49	>0.99
3	CDK1SA	>11 *vs* ⩽11	6.29	1.46	27.10	**0.014**
	pT stage	4 *vs* 3	1.29	0.38	4.40	0.690
4	CDK1SA	>11 *vs* ⩽11	6.52	1.51	28.14	**0.012**
	Sex	Female/male	0.48	0.18	1.25	0.130
5	CDK1SA	>11 *vs* ⩽11	6.57	1.51	28.54	**0.012**
	Age	(years)	1.01	0.98	1.05	0.470
6	CDK1SA	>11 *vs* ⩽11	5.6	1.287	24.42	**0.022**
	LN resected	>12 *vs* ⩽12	0.52	0.21	1.31	0.165
7	CDK1SA	>11 vs ⩽11	8.23	1.07	63.54	**0.043**
	CEA	(Serum level)	0.99	0.89	1.10	0.828
8	CDK1SA	>11 *vs* ⩽11	11.08	1.46	83.93	**0.020**
	Obstruction	Yes/no	0.68	0.19	2.39	0.545
9	CDK1SA	>11 *vs* ⩽11	9.85	1.29	75.32	**0.028**
	Perforation	Yes/no	0.00	0.00		0.988
10	CDK1SA	>11 *vs* ⩽11	11.43	1.51	86.73	**0.018**
	Lymphinvasion	Yes/no	0.22	0.63	7.73	0.219
11	CDK1SA	>11 *vs* ⩽11	11.44	1.51	86.73	**0.018**
	Angioinvasion	Yes/no	4.75	0.62	36.69	0.135
12	CDK1SA	>11 *vs* ⩽11	11.17	1.48	84.57	**0.019**
	BRAF	Mutated/WT	0.39	0.05	2.92	0.356

Abbreviations: CI=confidence interval; HR=hazard ratio; pT=tumour stage; WT=wild-type.

a 82 cases (32%) with missing value for stroma content. CKD1SA is not significant (*P*=0.180), however, in all other tests against confounding factors, CDK1SA achieved significance.
