# Specific activity of cyclin-dependent kinase I is a new potential predictor of tumour recurrence in stage II colon cancer

**DOI:** 10.1038/bjc.2012.38

**Published:** 2012-02-14

**Authors:** E C M Zeestraten, M Maak, M Shibayama, T Schuster, U Nitsche, T Matsushima, S Nakayama, K Gohda, H Friess, C J H van de Velde, H Ishihara, R Rosenberg, P J K Kuppen, K-P Janssen

**Correction to:** *British Journal of Cancer* (2011) **106**, 133–140; doi:10.1038/bjc.2011.504

Upon publication of this paper earlier in Volume 106, the authors noticed an error in Figure 2, whereby panels A and B were exact duplicates. The correct Figure is now shown below.

The authors would like to apologise for this error.

## Figures and Tables

**Figure 2:**
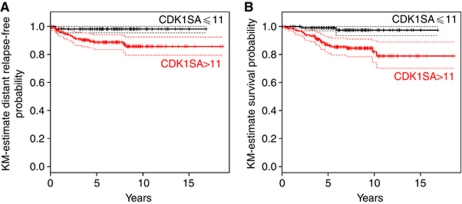
Analysis of distant metastasis-free survival and cause-specific survival. (**A**) Patients classified in the high-risk group (based on CDK1SA >11 maU eU^–1^) had a significantly worse distant metastasis event rate as compared with the low-risk group (HR=6.2, 95% CI: 1.45–26.9, *P*=0.0049; exact conditional Monte-Carlo *P*-value=0.029). (**B**) Patients classified in the CDK1SA-based high-risk group had a significantly lower cause-specific survival (HR=7.62, 95% CI: 1.80–32.2, *P*=0.001).

