# Supplementary material for: Specific activity of cyclin-dependent kinase I is a new potential predictor of tumour recurrence in stage II colon cancer
Source: Br J Cancer. 2011 Nov 22;106(1):133–40. doi: 10.1038/bjc.2011.504 (PMC3251853; doi:10.1038/bjc.2011.504)
Supplement: Supplementary Information [file bjc2011504x1.doc]

SUPPLEMENTARY DATA

**Specific activity of cyclin dependent kinase 1 predicts tumor recurrence in stage II colon cancer**

By Eliane.C.M. Zeestraten, Matthias Maak et al.

**Supplementary Materials Section.**

Repeatability and accuracy of the assay is monitored using lysate obtained from K562 cell, a human leukemia cell line, as a quality control (QC) material. True values of activities and expressions of CDK1 and CDK2 of QC material are determined in advance of the assay. Activities and expressions of CDKs of QC material were re-measured and monitored at the same time those of clinical sample were measured. Relative values were determined by dividing monitored value of QC material by true value of QC material, and were plotted on a Shewhart control chart. Mean values of relative activities of CDK1 and CDK2 of QC material were 98.8% and 96.8% (N=22). Mean values of relative expressions of CDK1 and CDK2 of QC material were 99.5% and 98.9% (N=25). Moreover, the Shewhart chart analysis did not show any systematic error in the distribution of relative values of QC material .

**Supplementary Figure 1** Distribution of specific activities of CDK1 and CDK1 for both study centers. Histograms of CDKSA distribution in samples from TUM, Germany (n=217) and LUMC, Netherlands (n=37), respectively. There was no significant difference in the distribution of CKD1SA (Mann-Whitney U test, p = 0.35), but the average of CDK2SA was significantly higher in the samples of LUMC (p = 0.012).

**Supplementary Figure 2** Bootstrap sampling analysis showing estimated distribution of optimal CDK1SA cut-off values for distant metastases risk discrimination. Green filled bars representing CDK1SA cut-off values 11 and 18.

**Supplementary Figure 3.** Prognostic performance of Microsatellite instability analysis. Significant difference in distant-metastasis free survival rate was not observed between MSS and MII-H cases (HR = 4.9: 95% CI: 0.64 to 37.7, p = 0.091).

**Supplementary Figure 4** Correlation between CDK1SA and microsatellite status. Distribution of CDK1SA of tumors which were determined either as MSI-High or microsatellite stable (MSS), indicated as box-whisker plot. CDK1SA expression levels in MSS cases were higher than in MSI-H cases, but differences did not attain significance, p=0.188.


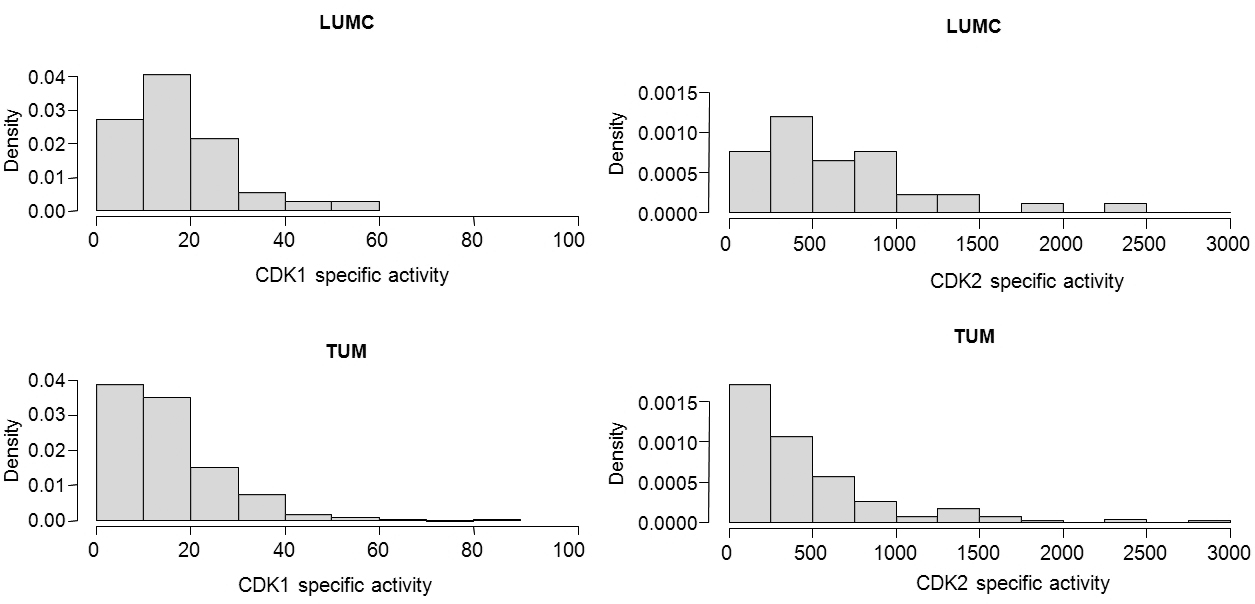


**Supplementary Figure 1**


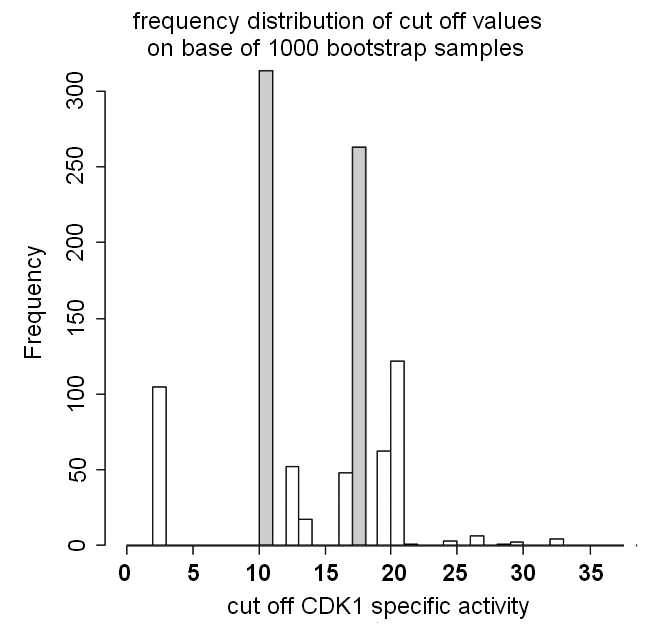


**Supplementary Figure 2**

**
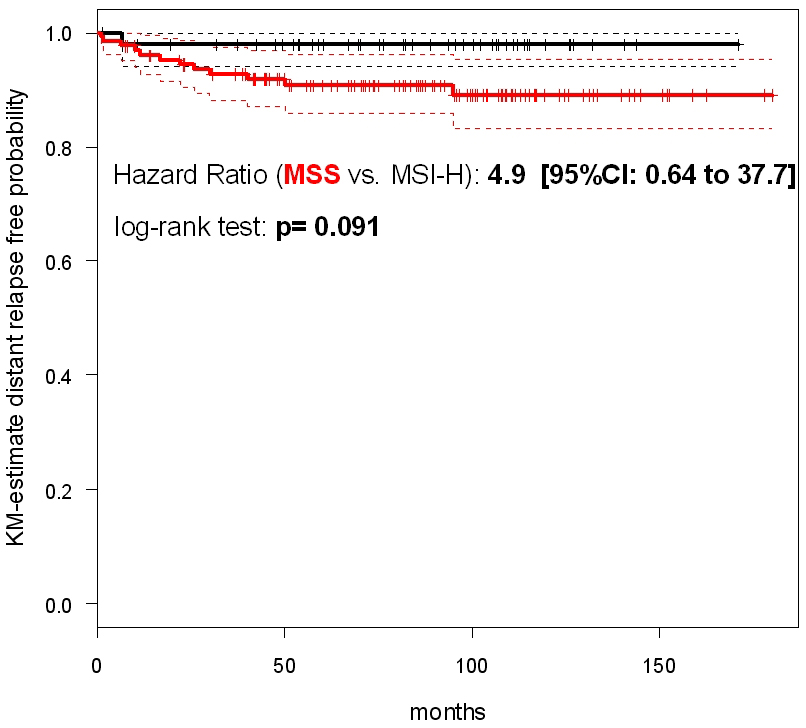
Supplementary Figure 3**


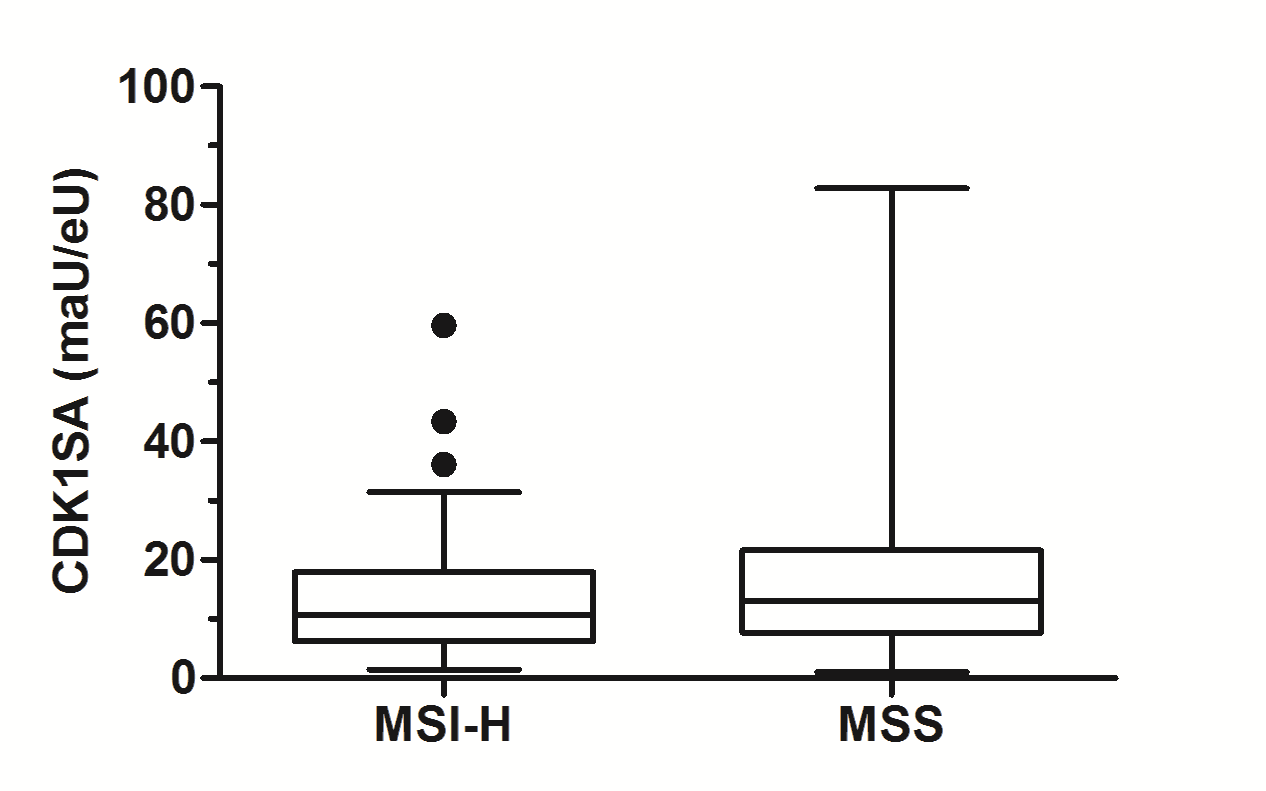


**Supplementary Figure 4**
